# Exploring vitamin D metabolism and function in cancer

**DOI:** 10.1038/s12276-018-0038-9

**Published:** 2018-04-16

**Authors:** Sang-Min Jeon, Eun-Ae Shin

**Affiliations:** 1 College of Pharmacy, Ajou University, Suwon, Gyeonggi-do 16499 Republic of Korea; 2 Research Institute of Pharmaceutical Science and Technology, Ajou University, Suwon, Gyeonggi-do 16499 Republic of Korea

## Abstract

Vitamin D, traditionally known as an essential nutrient, is a precursor of a potent steroid hormone that regulates a broad spectrum of physiological processes. In addition to its classical roles in bone metabolism, epidemiological, preclinical, and cellular research during the last decades, it revealed that vitamin D may play a key role in the prevention and treatment of many extra-skeletal diseases such as cancer. Vitamin D, as a prohormone, undergoes two-step metabolism in liver and kidney to produce a biologically active metabolite, calcitriol, which binds to the vitamin D receptor (VDR) for the regulation of expression of diverse genes. In addition, recent studies have revealed that vitamin D can also be metabolized and activated through a CYP11A1-driven non-canonical metabolic pathway. Numerous anticancer properties of vitamin D have been proposed, with diverse effects on cancer development and progression. However, accumulating data suggest that the metabolism and functions of vitamin D are dysregulated in many types of cancer, conferring resistance to the antitumorigenic effects of vitamin D and thereby contributing to the development and progression of cancer. Thus, understanding dysregulated vitamin D metabolism and function in cancer will be critical for the development of promising new strategies for successful vitamin D-based cancer therapy.

## Cancer: The significance of vitamin D metabolism

Altered vitamin D metabolism in cancer patients might help tumors progress, and understanding this effect could guide new anti-cancer therapies. Sang-Min Jeon and Eun-Ae Shin at Ajou University in South Korea review research on links between cancer and vitamin D metabolism. Vitamin D is best known for its role in building and maintaining healthy bones. Many epidemiological studies, however, suggest vitamin D deficiency is also involved in diseases affecting tissues other than bone. Sunlight promotes vitamin D formation, and low exposure to sunlight was found to be associated with increased incidence of cancer more than 80 years ago. Vitamin D deficiency has since been implicated in at least 18 types of cancer. The authors summarize the metabolic processes underlying the anti-cancer effects of vitamin D, and discuss ways to improve vitamin D-based therapies for cancer.

## Introduction

Vitamin D is a fat-soluble vitamin obtainable from the diet, as well as a seco-steroidal prohormone produced in the skin by ultraviolet B (UVB, 290–320 nm) from sunlight. Vitamin D, as a precursor of a potent steroid hormone, undergoes two-step metabolism in the liver and kidney to synthesize a biologically active form, calcitriol, which binds to the vitamin D receptor (VDR) to enable its diverse physiological functions^1,2^. The classical role of vitamin D is to regulate metabolism of calcium and phosphate, which is essential for bone remodeling. However, extensive research over the past decades has suggested that low sunlight exposure and vitamin D deficiency are also associated with the increased risk of many other extra-skeletal diseases such as cancer^3–6^.

The first observation of an inverse correlation between sunlight exposure and overall cancer incidence and mortality in North America was published almost 80 years ago^7,8^. Later, in 1980 and 1992, the first epidemiological studies linking low sunlight exposure and high risk of colon and prostate cancers were reported, respectively, which suggested that vitamin D as a surrogate for sunlight exposure may protect against colon and prostate cancer risk^9,10^. Since then, many epidemiological studies have supported and extended the UVB–vitamin D–cancer hypothesis in 18 different types of cancers^11^. The hypothesis has been further supported by studies showing the direct association between vitamin D and cancer risk. Several lines of population-based studies revealed an inverse correlation between serum 25-hydroxyvitamin D (25(OH)D) levels and high risk of colon^12^, breast^13^, prostate^14,15^, gastric, and other cancers^16^. Moreover, there are strong evidences from several cell culture and animal studies to support the antitumorigenic effects of vitamin D^6,17,18^. As such, it is now becoming evident that deficiency of vitamin D can contribute to the development and progression of many types of cancers, and thus maintenance of sufficient serum vitamin D levels could be beneficial for prevention and treatment of cancer.

Because the numerous epidemiological and experimental data indicate the beneficial role of vitamin D in the prevention and treatment of several types of cancers, clinical use of calcitriol or vitamin D analogs has been investigated^17^. However, hypercalcemia, the major toxic effect of vitamin D, has strongly limited its clinical applications^19,20^. Moreover, accumulating data suggest that cancer cells employ several mechanisms that reduce cellular calcitriol levels, as well as diminish its function to protect themselves from the antitumorigenic effects of vitamin D^17,18^. Thus, understanding how vitamin D metabolism and signaling are dysregulated in cancer will help develop efficient therapeutic strategies to overcome such limitations of using vitamin D or its analogs for clinical purpose.

In this review, we provide an overview of vitamin D metabolic pathways and summarize antitumorigenic functions and mechanisms of vitamin D. In addition, we discuss how vitamin D metabolism and function are dysregulated in cancer to promote resistance to the antitumorigenic effect of vitamin D. Finally, we discuss future directions to overcome the limitations of and improve vitamin D-based cancer therapy.

## Vitamin D metabolism

**Fig. 1:**
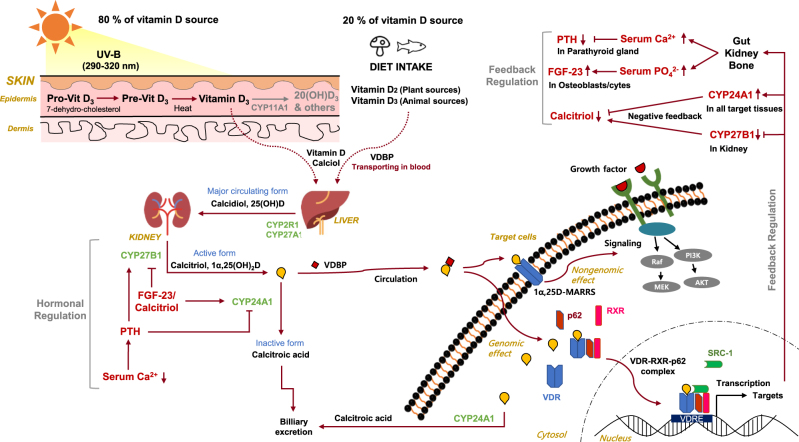
**Overview of vitamin D metabolism**

**Fig. 2:**
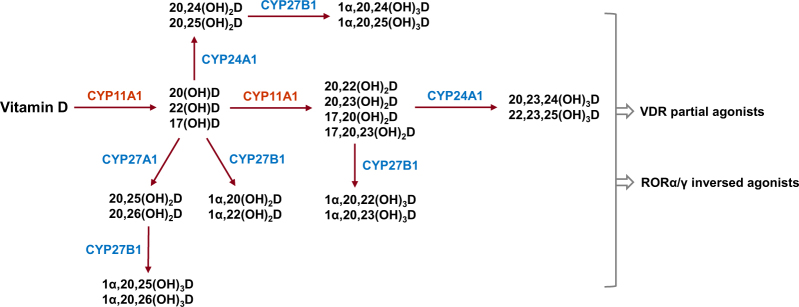
**The metabolites generated from alternative vitamin D metabolic pathway**

Vitamin D is a prohormone that needs to be metabolized to biologically active products that bind to their cognate nuclear receptors for regulation of diverse physiological processes^2^. In this section, the classical and alternative vitamin D metabolic pathways, and hormonal regulation of vitamin D metabolism, are discussed and summarized in Figs. 1 and 2.

### Classical metabolic pathway

There are two major isoforms of vitamin D, vitamin D_2_ (ergocalciferol), and vitamin D_3_ (cholecalciferol)^21,22^. Vitamin D_2_ is synthesized from ergosterol by UVB radiation in plants, yeasts, and fungi and can be ingested in a diet containing plant source foods, such as mushrooms. Vitamin D_3_ is synthesized from 7-dehydrocholesterol by UVB radiation in the epidermis of skin and can be also ingested in a diet of animal source foods, such as cod liver oil. Vitamin D (both vitamin D_2_ and D_3,_ calciol) originating from either the diet or the skin binds to vitamin D-binding protein (VDBP) in circulation and is first delivered to the liver.

In the liver, vitamin D is metabolized by vitamin D 25-hydroxylase (CYP2R1 and CYP27A1) to 25(OH)D (calcidiol), which is the major circulating form of vitamin D in serum^23,24^. 25(OH)D is further metabolized by 25(OH)D 1α-hydroxylase (CYP27B1) mainly in the proximal tubule of the kidney to 1α,25-dihydroxyvitamin D [1α,25(OH)_2_D, calcitriol], which is the most biologically active form of vitamin D^23,24^. Calcitriol then enters the circulation and, after binding to VDBP, is delivered target tissues such as intestine, bone, and kidney, where vitamin D is known to regulate absorption, mobilization, and reabsorption, respectively, of calcium and phosphate^21^. After being produced, the levels of both calcidiol and calcitriol are tightly regulated by 25(OH)D 24-hydroxylase (CYP24A1), which is the primary vitamin D inactivating enzyme catalyzing hydroxylation at C-24 and C-23 of both calcidiol and calcitriol^23,24^. The 24-hydroxylase pathway produces biologically inactive biliary excreted calcitroic acid, whereas the less known 23-hydroxylase pathway produces 1,25–26,23 lactone, whose relative activity by CYP24A1 catalysis shows species difference^23,24^. The importance of CYP24A1-driven calcitriol inactivation was underscored by the finding of impaired intramembranous bone mineralization and hypercalcemia in *Cyp24a1* knockout mice, leading to perinatal death in 50% of the mice^25,26^. Interestingly, the defect was normalized in *Cyp24a1* and *VDR* double knockout mice, suggesting that increased calcitriol levels, but not the absence of 24- or 23-hydroxylated vitamin D metabolites, were responsible for the defective phenotype^25^.

In target tissues, calcitriol binds to VDR, a member of the nuclear receptor family of ligand-activated transcription factors and which induces both genomic and non-genomic regulation of downstream targets involving diverse biological functions^2,27,28^. In the genomic pathway, calcitriol binds to cytosolic VDR, which promotes phosphorylation of VDR, heterodimerizaion with retinoid-X receptor (RXR), and then nuclear translocation of the complex^29^. The calcitriol–VDR–RXR complex binds to vitamin D response element (VDRE) in the promoter region of its target genes and recruits transcriptional coactivators or co-repressors to regulate mRNA expression of target genes and thus regulating a variety of their functions, including calcium and phosphate metabolism. Interestingly, a recent study showed that autophagy adaptor protein p62/SQSTM1 plays a key role in heterodimerization and recruitment of the VDR–RXR complex to target genes by directly binding to VDR and RXR in hepatic stellate cells^30^. In the non-genomic pathway, calcitriol binds to membrane bound VDR, which is identified as 1,25D-membrane-associated, rapid response steroid-binding protein (1,25D-MARRS); this interaction then induces acute changes in cell signaling pathways, including calcium and mitogen-activated protein kinase (MAPK) signaling, through direct protein–protein interaction with intracellular signaling molecules involved in certain phenotypic functions^27,31^. To elicit the full spectrum of biological functions of calcitriol, both genomic and non-genomic pathways should be investigated.

### Alternative metabolic pathway

Recently, an alternative pathway of vitamin D metabolism via CYP11A1, also known as a cytochrome P450 side chain cleavage (P450scc) enzyme, has been reported^32–34^. Originally, CYP11A1 was known to catalyze the first rate-limiting step of steroidogenesis in steroidogenic organs by inducing hydroxylation of cholesterol at C-22 and C-20 followed by cleavage of the bond between C-20 and C-22 to generate pregnenolone, the common precursor of steroid hormones^35^. However, CYP11A1 is emerging as a new vitamin D-metabolizing enzyme by the finding of the expression of CYP11A1 in peripheral tissues such as skin and gastrointestinal (GI) tract, and that vitamin D is an alternative substrate to cholesterol for the enzyme^36,37^. CYP11A1-mediated metabolism of vitamin D involves sequential hydroxylation, predominantly at C-20 or C-22, without the cleavage of the side chain. The main vitamin D metabolites resulting from a single hydroxylation by CYP11A1 include 20(OH)D, 22(OH)D, and 17(OH)D^32^. These are further hydroxylated by CYP11A1 to generate 20,23(OH)_2_D, 20,22(OH)_2_D, 17,20(OH)_2_D, and 17,20,23(OH)_3_D. Moreover, the major product of this pathway, 20(OH)D, can also serve as a substrate for CYP27A1 and CYP24A1, with CYP27A1 hydroxylating it at C-25 or C-26, and CYP24A1 hydroxylating it at C-24 or C-25. These products can be further hydroxylated at C-1α by CYP27B1 generating corresponding trihydroxy-vitamin D metabolites, except for 17,20,23(OH)_3_D, which is the final vitamin D metabolite generated by the CYP11A1-driven metabolic pathway. Altogether, it has been estimated that this alternative metabolic pathway can produce more than 21 hydroxymetabolites of vitamin D (Fig. 2)^32^.

The products of CYP11A1, such as 20(OH)D and its hydroxymetabolites, induce anti-proliferation, differentiation, and anti-inflammation in skin cells comparable or better than that of calcitriol^38,39^. Moreover, these metabolites enhance defense mechanisms against UVB-induced DNA damage and oxidative stress and, importantly, elicit anticancer properties in a cell line-dependent manner^37^. Interestingly, the products of CYP11A1, such as 20(OH)D and 20,23(OH)D, have been shown to function as partial or biased agonists of VDR^32^. For example, these metabolites do not induce calcemic effects or the expression of CYP24A1 in pharmacological concentrations that activate VDR, which can normally be seen in response to calcitriol treatment.

In addition to VDR, α and γ isoforms of retinoid-related orphan receptors (RORα, RORγ), members of the nuclear receptor family of ligand-dependent transcription factors, have been shown to function as novel receptors for CYP11A1-derived vitamin D metabolites such as 20(OH)D and 20,23(OH)D^32,40,41^. RORs play key roles in the regulation of many physiological processes including immune and metabolic pathways and are implicated in many pathologies such as cancer, autoimmune diseases, and metabolic syndrome^41^. Interestingly, the CYP11A1-derived vitamin D metabolites act as inverse agonists of RORα and RORγ, which inhibits their transcriptional activity. For example, 20(OH)D and 20,23(OH)D can suppress the transcription of RORα and RORγ target genes such as *Bmal1* and *G6Pase*, respectively^40^. Thus, the pleiotropic and diverse effects of vitamin D could be attributed not only to the effects of the calcitriol–VDR pathway, but also to those of CYP11A1-derived vitamin D metabolites–VDR or –RORα/γ pathways. Further work should be conducted to define their specific contributions to the broad spectrum of vitamin D’s effects in health and diseases.

### Hormonal regulation of classical vitamin D metabolism

Interestingly, calcitriol, as a hormone, tightly regulates vitamin D metabolism in a negative feedback mechanism^42,43^. Importantly, the vitamin D inactivating enzyme, CYP24A1, is among the strong transcriptional targets of the calcitriol–VDR–RXR complex. The promoter region of CYP24A1 contains two VDREs approximately 150 and 250-bp upstream of the transcriptional start site, which drives strong induction of CYP24A1 by calcitriol^44^. In addition, calcitriol can also induce CYP24A1 expression by recruiting histone H4 acetyltransferases and RNA polymerase II to a site approximately 50- to 70-kb downstream of the human *CYP24A1* gene^45^. Thus, the levels of calcidiol and calcitriol can be tightly regulated by calcitriol-driven expression of CYP24A1 in the kidney. Moreover, calcitriol also inhibits the transcription of *CYP27B1* in the kidney through complex mechanisms involving epigenetic modifications of its promoter region^46^.

In addition to the negative feedback regulation by calcitriol, vitamin D metabolism is also regulated by two hormones, parathyroid hormone (PTH) and fibroblast growth factor-23 (FGF-23), both of which play major roles in maintaining calcium and phosphate homeostasis^47–49^. PTH is secreted by the parathyroid gland in response to low serum calcium levels, as sensed by calcium-sensing receptors (CaSRs) expressed on the surface of parathyroid cells^48^. PTH stimulates renal expression of CYP27B1 by mechanisms involving increased cAMP-dependent transcription or upregulation of nuclear orphan receptor NR4A2-dependent transcription, leading to the increase in calcitriol production^50,51^. Although increased calcitriol can induce its own degradation by inducing the expression of CYP24A1, PTH can sustain calcitriol levels by inducing the degradation of *CYP24A1* mRNA through activation of the cAMP–PKA pathway in the kidney^52,53^. The resulting high calcium levels by sustained calcitriol induction can negatively regulate the secretion of PTH through binding to CaSRs in the parathyroid gland as a negative feedback mechanism^54^. FGF-23 is secreted by osteoblasts and osteocytes in response to both high serum phosphate and calcitriol levels^47^. FGF-23 facilitates the excretion of phosphate by inhibiting the expression of sodium–phosphate cotransporter 2 (NPT2) located at the apical membranes of proximal renal tubules through binding to FGF receptor–Klotho complexes in cell membranes. In addition, FGF-23 reduces serum calcitriol levels by inhibiting the expression of CYP27B1, whereas stimulating the expression of CYP24A1 in the kidney, although the mechanisms remain to be elucidated^55–57^.

## Anticancer properties of Vitamin D

**Fig. 3:**
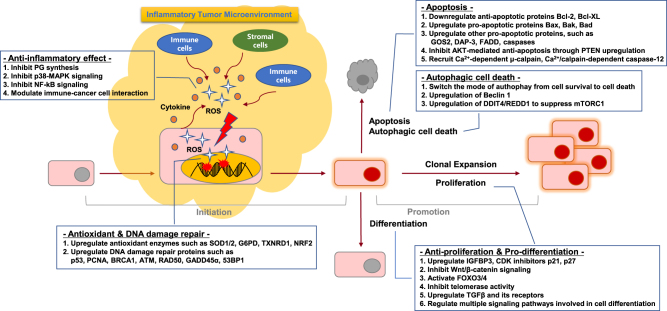
**Anticancer properties of vitamin D**

As the beneficial effects of vitamin D in cancer prevention and treatment have been observed in epidemiological and preclinical studies, diverse mechanisms have been proposed to explain its anticancer effects. Accumulating data suggest that vitamin D can regulate the entire process of tumorigenesis, from initiation to metastasis and cell–microenvironment interactions^17^. These mechanisms include regulation of cell behaviors such as proliferation, differentiation, apoptosis, autophagy, and epithelial–mesenchymal transition (EMT), and modulation of cell–microenvironment interactions such as angiogenesis, antioxidants, inflammation, and the immune system. As the role of vitamin D in cancer has been extensively reviewed in other studies^6,17^, here, we focus on its versatile roles in tumor initiation and promotion stages, which are summarized in Fig. 3.

### Initiation stage: role in anti-inflammation, antioxidant defense and DNA damage repair

Tumor initiation is a process that introduces irreversible genetic mutations in normal cells, consequently inducing transformation. A body of data supports that vitamin D plays a key role in preventing the initiation stage by exerting anti-inflammatory and antioxidant defenses and DNA damage repair processes^6,17^.

### Anti-inflammation

Chronic inflammation is a low-grade, prolonged inflammatory response resulting in progressive destruction and regeneration of tissues by reactive oxygen species (ROS) and cytokines secreted at the site of inflammation. It is now well-accepted that chronic inflammation is one of the main contributors to the initiation of tumorigenesis^58^. Accumulating data suggest that vitamin D exerts anti-inflammatory effects via at least four mechanisms.

First, calcitriol inhibits the prostaglandin (PG) pathway involved in pro-inflammatory responses through inhibition of the expression of cyclooxygenase-2 (COX-2) and PG receptors, and degradation of PGs^59^. In prostate cancer cells, calcitriol reduces the expression levels of COX-2 and that of PG receptors EP2 and FP, whereas it increases the expression of 15-hydroxyprostaglandin-dehydrogenase (15-PGDH), a NAD^+^-dependent enzyme responsible for the degradation of PGE2^59,60^. In addition, the decrease of *COX-2* mRNA expression and PGE2 production has also been reported in calcitriol-treated breast cancer cells^61^. Consistently, the expression of VDR is inversely correlated with that of COX-2 in malignant breast cell lines and ovarian cancer tissues^62,63^, supporting the role of the calcitriol–VDR axis in suppressing the expression of COX-2 and production of PGs.

Second, vitamin D can suppress the p38 MAPK-mediated pro-inflammatory signaling pathway. In both normal prostate epithelial cells and prostate cancer cells, calcitriol inhibits the production of pro-inflammatory cytokines such as IL-6 by inducing the expression of MAPK phosphatase-5 (MKP-5), which prevents the phosphorylation and activation of p38 MAPK^64^. In addition to prostate cells, calcitriol also inhibited Lipopolysaccharides (LPS)-induced production of interleukin-6 (IL-6) and tumor necrosis factor (TNF)-α through the induction of MKP-1 in human monocytes and murine macrophages^65^.

Third, calcitriol can also inhibit the nuclear factor kappa B (NFκB) signaling pathway through several mechanisms. Calcitriol suppresses the phosphorylation of both AKT and its downstream target I kappa Bα (IκBα) in macrophages through upregulation of thioesterase superfamily member 4 (THEM4), an AKT modulator protein leading to the inhibition of NFκB and COX-2 expression^66^. In fibroblasts, calcitriol augmented the protein stability of IκBα, and also induced the binding of VDR to IκBα kinase (IKK), preventing its phosphorylation and activation and thereby inhibiting the nuclear translocation of the p65 subunit of NFκB^67,68^.

Fourth, vitamin D can regulate the interaction between immune and cancer cells to suppress the production of pro-inflammatory cytokines. Co-culture experiments using peripheral blood mononuclear cells (PBMCs) and colon cancer cells revealed that vitamin D treatment significantly decreased the production of pro-inflammatory cytokines such as TNF-α, IL-6 and, to a lesser extent, IL-10, supporting the anti-inflammatory effects of vitamin D in tumor microenvironment^69^.

### Antioxidant defense and DNA damage repair

ROS play a key role in many aspects of tumorigenesis by promoting DNA mutation, cell proliferation, and cell death that also provokes pro-inflammatory responses. Thus, maintaining antioxidant defense systems should be a critical step in preventing tumor development. Accumulating data suggest that vitamin D can protect from oxidative stress-induced DNA damage by promoting antioxidant defenses^70,71^. It was shown in mice that DNA damage induced by oxidative stress was elevated in colon epithelial cells of VDR-knockout mice^72^. Moreover, the treatment of rats with calcitriol markedly reduced the levels of malondialdehyde, the end product of lipid peroxidation causing DNA damage^73^. In line with this, daily supplementation of vitamin D in humans reduced oxidative DNA damage suggesting that vitamin D may protect against oxidative stress-induced DNA damage in humans^74^.

Vitamin D-mediated protection from ROS-induced DNA damage can be attributed to its role in inducing the expression of numerous enzymes involved in ROS detoxification. Calcitriol induced the expression of superoxide dismutase 1 (SOD1) and 2 (SOD2) in prostate epithelial cells (PECs) and in androgen-sensitive prostate cancer cells (LNCaP), respectively^75,76^. In addition, calcitriol induced the expression of thioredoxin reductase 1 (TXNRD1), which reduces thioredoxin for its antioxidant function in prostate and breast cancer cells^75,77^ and glucose-6-phosphate dehydrogenase (G6PD), which produces NADPH for glutathione (GSH) regeneration in prostate and ovarian cancer cells^78,79^. Moreover, NF-E2-related factor-2 (NRF2), a transcription factor that increases the expression of a diverse array of antioxidant enzymes was shown to be regulated by vitamin D through either increase in its expression, nuclear translocation, or decrease in KEAP1-mediated degradation^80–82^. As NRF2 is known as the master regulator of the expression of antioxidant enzymes, this can be a potential mechanism by which vitamin D can induce antioxidant enzymes and exert antioxidant defenses^83^.

In addition to preventing DNA damage by augmenting antioxidant capacity, vitamin D can also directly regulate DNA damage repair processes^71^. Studies showed that vitamin D increases the expression of genes involved in DNA damage repair such as *p53*, proliferating cell nuclear antigen (*PCNA*), and breast cancer 1 (*BRCA1*) in breast cancer cells^77^ and ataxia-telangiectasia mutated (*ATM*) and recombinant DNA repair protein (*RAD50*) in PECs^84^, and growth arrest and DNA damage-inducible α (*GADD45*α) in SCC and ovarian cancer cells^85,86^. Vitamin D can also prevent the degradation of p53-binding protein 1 (53BP1) mediated by cysteine proteinase Cathepsin L, a lysosomal endopeptidase, in breast cancer^87^. Thus, vitamin D could be critical for preventing genetic mutations at the tumor initiation stage by inducing anti-inflammatory, antioxidant, and DNA damage repair functions.

### Promotion stage: role in cell proliferation/differentiation and apoptosis/autophagy

Even in cells with established genetic mutations during the initiation stage, vitamin D still elicits anticancer properties by blocking the tumor promotion stage through inhibition of cell proliferation, induction of cell differentiation, and cell death.

### Cell proliferation and differentiation

Calcitriol was shown to elicit anti-proliferation and pro-differentiation properties both in normal and malignant cells^88^. In addition to calcitriol, novel vitamin D metabolites produced by CYP11A1 such as 20(OH)D_3_, 20(OH)D_2_, 1,20(OH)_2_D_3_, and 20,23(OH)_2_D_3_, were also shown to inhibit cell proliferation and induce cell differentiation through VDR, which is comparable to those of calcitriol, but with less calcemic effects^32,39^. The anti-proliferative property of vitamin D is mediated by multiple mechanisms including the regulation of growth factors, cell cycle, and signaling pathways. Vitamin D increases the expression of insulin-like growth factor (IGF)-binding protein 3 and the cyclin-dependent kinase (CDK) inhibitors, p21 and p27, whereas inhibiting the expression of CDK2, leading to inhibition of IGF-1- and IGF-2-stimulated cell proliferation and cell cycle progression^88^. In addition, calcitriol inhibits the Wnt/β-catenin signaling pathway by decreasing the formation of transcription factor 4-β-catenin, (TCF4-b)–catenin complexes or increasing the expression of the Wnt antagonist, Dickkopf-1 (DKK-1)^89,90^. Moreover, vitamin D also activates transcription factors forkhead box O3/4 (FoxO3/4), which trigger transcription of target genes involved in cell cycle arrest and anti-proliferation, by inducing their deacetylation and dephosphorylation in neuroblastoma cells^91^. Vitamin D was also shown to inhibit telomerase activity by reducing the expression of telomerase reverse transcriptase (TERT) via microRNA-498^92^ and to induce the expression of transforming growth factor β (TGFβ), as well as its receptors, leading to inhibition of cell growth^93,94^. The induction of differentiation by vitamin D is associated with the anti-proliferative properties and regulation of diverse intracellular signaling pathways, such as phosphatidylinositol 3 kinase/AKT, MAPK, NF-kB, and Ca^2+^ signaling^88^.

### Apoptosis and autophagy

Vitamin D-induced apoptosis is mainly mediated by both downregulation of the anti-apoptotic proteins Bcl-2 and Bcl-XL, and upregulation of pro-apoptotic proteins Bax, Bak, and Bad^95^. In addition, induction of apoptosis by upregulation of other pro-apoptotic proteins such as G0-G1 switch 2 (GOS2), death-associated protein (DAP-3), Fas-associated death domain (FADD), and caspases was reported^77,96^. Vitamin D can also inhibit the AKT-mediated anti-apoptotic signaling pathway by increasing phosphatase and tensin homolog (PTEN) expression^97^. Moreover, vitamin D can also initiate apoptotic events by recruiting Ca^2+^-dependent apoptotic effectors such as Ca^2+^-dependent μ-calpain and Ca^2+^/calpain-dependent caspase-12^98^.

Autophagy is a catabolic process that plays a critical role in both cell survival and apoptosis-independent cell death. Interestingly, accumulating data suggest that vitamin D can switch the mode of autophagy from cell survival to cell death in cancer cells. Vitamin D was shown to elicit cytotoxic or cytostatic autophagy, which sensitized radiotherapy commonly inducing cytoprotective autophagy^99,100^. Vitamin D-induced autophagic cell death can be attributed to the upregulation of *beclin 1*, an autophagy-related gene^101^. In addition, CDK inhibitors may be involved in vitamin D-mediated autophagic cell death because this was enhanced by loss of p19 or attenuated by loss of p27^102^. Moreover, vitamin D can also induce autophagy by inducing the expression of DNA damage-inducible transcript 4 (*DDIT4*), also known as regulated in development and DNA damage response 1 (*REDD1*), an inhibitor of mechanistic target of rapamycin complex 1 (mTORC1), which is known to suppress autophagy^103,104^.

## Dysregulation of vitamin D activity in cancer

**Fig. 4:**
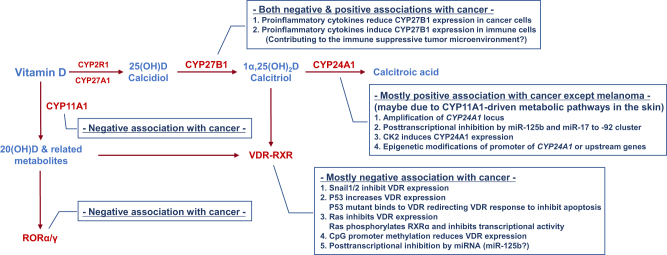
**Dysregulation of vitamin D metabolism in cancer**

Classically, it has been considered that the calcitriol producing enzyme CYP27B1 is expressed in the kidney, and that VDR and CYP24A1, which mediate calcitriol function and degradation, are expressed in certain target tissues such as intestine, bone, and kidney. However, accumulating data suggest that these proteins are widely expressed in many tissues other than the kidney or the target tissues^105,106^ suggesting that most cells could be targets of vitamin D and that calcitriol levels could be locally regulated to exert fine-tuned tissue-specific functions. Importantly, this local regulation of vitamin D activity is dysregulated in many cancer cells, which contributes to the resistance to vitamin D-based cancer therapy^3,17,18,43^. In this section, our current understandings about the dysregulation of vitamin D metabolism and function in cancer are discussed and summarized in Fig. 4.

### VDR/RXRα

VDR is widely expressed in most cell types, but the expression is progressively reduced during dedifferentiation and tumor progression in many cancer types. Comparing VDR expression levels in normal, benign, and malignant tissues of skin, breast, ovarian, and prostate revealed a negative correlation between VDR expression and tumor malignancy^107–112^. Importantly, high VDR expression is significantly associated with reduced risk of lethal prostate cancer progression and cancer death^112^ and also nuclear VDR expression is associated with better overall survival in lung cancer patients^113^. Consistently, a recent study showed that reduced VDR protein expression is observed in urothelial bladder cancer and is associated with poor prognosis of patients^114^. These observations suggest that VDR expression may be useful as a valuable early diagnosis biomarker for high-risk populations. Furthermore, vitamin D could be critical in preventing cancer progression and thus cancer cells may actively counteract the tumor-suppressive effects of vitamin D by developing multiple mechanisms to abrogate VDR expression, as well as its activity.

First, snail family transcriptional repressor (Snail) that is overexpressed in several cancers and involved in EMT, tumor invasion, and metastasis was shown to inhibit VDR expression in cancer^115^. It was shown that Snail1 and Snail2 can bind to E-boxes in the proximal promoter region of the *VDR* gene to recruit co-repressors that inhibit the transcription of *VDR* in colon and breast cancer cells^116,117^. Second, tumor-suppressor p53, which is lost or mutated in almost half of all tumors, can increase the transcription of VDR^118^. Interestingly, it was shown that cancer-associated p53 mutant cells can also regulate VDR responses by directly binding to VDR and redirecting the VDR-mediated transcriptional program to protect cancer cells from apoptosis^119^. Third, constitutively active mutations of the *Ras* oncogene found in many cancers suppress VDR expression in tumors. The expression of *K-Ras* mutants in human colon cancer cells and *H-Ras* mutants in mouse colon and rat intestinal epithelial cells inhibit calcitriol-dependent VDR activation by suppressing *VDR* transcription^120^. In addition to reducing VDR expression, *H-Ras* and *K-Ras* mutations expressed in keratinocytes and PEC lines, respectively, suppressed *VDR* transcriptional activity by inducing phosphorylation of RXRα, which impairs the recruitment of co-activator SRC-1 to RXRα^121,122^. Forth, epigenetic silencing of *VDR* has been reported in cancer. CpG island methylation in the *VDR* promoter region was associated with reduced expression of VDR in colon and breast cancer cells^123,124^. Moreover, DNA methyltransferase (DNMT) inhibitor induced VDR expression and enhanced the anti-proliferative effect of calcitriol in breast cancer cells^123^. Finally, the involvement of microRNA (miRNA) was reported to control VDR expression in cancer^125,126^.

### CYP27B1

As calcidiol is the major circulating form of vitamin D, cells that express CYP27B1 may increase the local concentration of calcitriol further than do cells only dependent on the systemic calcitriol produced in the kidney. Similar to VDR, the expression of CYP27B1 is inversely correlated with the progression of tumors of lung, prostate, colon, parathyroid, and skin^108,127–132^, suggesting that local production of calcitriol in those tissues could be important for cancer prevention. Interestingly, a recent study showed that pro-inflammatory cytokines such as IL-6 and TNF-α downregulated the expression of CYP27B1 in colon cancer cells^133^, suggesting that the pro-inflammatory tumor microenvironment could be a contributing factor for decreased CYP27B1 levels during tumor progression. However, the molecular mechanisms responsible for the progressive reduction of CYP27B1 expression during cancer progression are largely unknown. In contrast to the negative association in those cancer types, a positive association was reported in thyroid cancer^134^ and conflicting results were also reported for breast^110,135^ and renal cancers^136,137^. Moreover, in contrast to lung cancer cells, CYP27B1 expression in alveolar macrophages from lung cancer patients showed positive association with cancer progression^138^. This could be explained in part by the finding that pro-inflammatory cytokines such as IFN-γ and TNF-α, and agonists of Toll-like receptors (TLRs) upregulated the expression of CYP27B1 in monocytes, macrophages, and dendritic cells^139,140^ suggesting that the pro-inflammatory tumor microenvironment could be a contributing factor for increased CYP27B1 expression in immune cells, which is opposite to the finding in colon cancer cells^133^ mentioned above. This change in CYP27B1 expression and vitamin D metabolism in immune cells may contribute to the immune-suppressive tumor microenvironment.

### CYP24A1

Given that CYP24A1 is an enzyme that degrades calcidiol and calcitriol, it is likely that cancer cells may upregulate CYP24A1 expression to reduce local concentrations of calcitriol, which is similar to the reduction of CYP27B1 in some cancers as discussed above. Indeed, *CYP24A1* has been found to be amplified in breast cancer and proposed as an oncogene^141^. Consistently, CYP24A1 expression is correlated with the advanced stages of colon, prostate, breast, and lung cancers, inducing resistance to vitamin D-based therapy^110,129,142–146^. In addition, overexpression of CYP24A1 has been also reported in many other cancer types including ovarian, cervical, squamous cell, and basal cell carcinoma^147–149^. Moreover, CYP24A1 expression is associated with poor prognosis in lung, colon, and esophageal cancer^145,150,151^. These findings suggest that cancer cells can evade the anticancer effects of calcitriol by inducing the expression of CYP24A1, which reduces the local concentration of calcitriol. Supporting the oncogenic role of CYP24A1, the inhibition of CYP24A1 suppressed tumor growth and potentiated the antitumorigenic effects of calcitriol in breast and lung cancers, suggesting that CYP24A1 could be a promising therapeutic target^152–154^. In contrast to the positive association in those cancer types, some conflicting data have been reported for prostate cancer^146,155^ and even an inverse correlation between CYP24A1 expression and tumor progression has been reported in melanoma^156^, which, here, is discussed later.

Although CYP24A1 expression is induced by calcitriol–VDR activation via negative feedback regulation, the high level of CYP24A1 expression observed in cancer cells is unlikely to be mediated by VDR activation because, as mentioned above, VDR expression and activity are downregulated in most cancer. This suggests that the overexpression of CYP24A1 in many cancer cells may not be the result of normal physiological processes regulated by calcitriol–VDR-dependent mechanisms. Currently, at least four mechanisms responsible for the induction of CYP24A1 in cancer have been proposed. First, as mentioned above, overexpression of CYP24A1 in breast cancer is associated with the amplification of chromosomal locus 20q13.2–20q13.3 containing the *CYP24A1* gene, which has been also observed in other cancers, including colon malignancies^141,157^. Interestingly, the amplification of CYP24A1 was detected only in malignant but not benign colon tumors, suggesting that CYP24A1 overexpression, and thereby inactivation of calcitriol, could be critical for tumors to progress to the malignant status^158^. Second, post-transcriptional regulation by miRNAs is associated with the CYP24A1 overexpression in cancer. MiR-125b, which is frequently downregulated in many cancers, was shown to bind to the 3′-UTR of *CYP24A1* mRNA and thereby inhibit its expression^159^. In addition, CYP24A1 expression levels were inversely correlated to miR-125b levels in breast cancer tissues^159^, suggesting that low levels of miR-125b may account for CYP24A1 overexpression in cancer. Moreover, a recent study showed that the miR-17 to -92 cluster also regulates CYP24A1 expression in lung cancer cells^160^. These authors showed that the inhibitor miR-92a, which reduces levels of the miR-17 to -92 primary transcripts, diminishes the level of CYP24A1 expression in p53-depleted lung cancer cells^160^. Third, the serine/threonine protein kinase casein kinase 2 (CK2) signaling pathway induces CYP24A1 overexpression in prostate cancers^161^. Interestingly, overexpression of CK2 has been observed in many cancers, including prostate cancer, and correlates with poor clinical outcomes^162^. Finally, epigenetic regulation involving the CYP24A1 promoter region contributes to the altered expression of CYP24A1 in cancers. It has been shown that the expression of CYP24A1 is inversely correlated with the methylation of the CYP24A1 promoter in lung and prostate cancer^155,163^. Consistently, the inhibition of DNMT or histone deacetylase (HDAC) increased the expression of CYP24A1 in lung and colon cancer^163^. Interestingly, however, the induction of CYP24A1 by the inhibition of epigenetic changes in colon cancer could be an indirect effect via regulation of genes operating upstream of *CYP24A1* suggesting the complexity of epigenetic regulation of CYP24A1 expression in cancer^164^.

### CYP11A1 and RORα/γ

The finding of unexpected expression patterns of CYP24A1 in melanoma proposed that, in context of the dominant CYP11A1-driven alternative vitamin D metabolism pathway in the skin, CYP24A1 can produce biologically active tumor-suppressive vitamin D metabolites rather than degrading calcitriol^39,156,165^. Interestingly, 20(OH)D produced by CYP11A1 can be hydroxylated by CYP24A1 to 20,24(OH)_2_D and 20,25(OH)_2_D, which are more potent in suppressing melanoma growth than calcitriol and 20(OH)D^165^. Consistently, the expression of RORα and RORγ, the receptors of the 20-hydroxylated vitamin D metabolites, are negatively correlated with melanoma progression and positively correlated with prognosis^166^. Supporting this tumor-suppressive role of CYP11A1-RORα/γ pathway in the skin, a recent study showed that CYP11A1 is also significantly downregulated in many other cancer types, including colon, kidney, liver, lung, prostate, and uterine corpus endometrial carcinoma^167^. In addition, the expression of CYP11A1 is also reduced in prostate cancer bone metastases^168^. Consistently, the expression of RORα and RORγ are positively associated with prognosis in patients with breast, lung, or liver cancer^169–172^. This association between the CYP11A1-RORα/γ pathway and tumor progression suggests that activation of the alternative vitamin D metabolism pathway could be a novel preventive, as well as therapeutic, strategy for cancer.

## Vitamin D-based cancer therapy: future directions

Although randomized clinical trial data are still lacking, several epidemiological, clinical, preclinical, and *in vitro* experimental data strongly suggest that the activation of vitamin D signaling could be a promising strategy for prevention, as well as treatment of many types of cancer. As such, several therapeutic interventions targeting dysregulated vitamin D metabolism or activity have been investigated and developed for cancer therapy^17^. However, there are some potential limitations of vitamin D-based cancer therapy, which should be taken into consideration to design better therapeutic strategies.

One potential caveat of systemic activation of vitamin D signaling would be the high risk of hypercalcemia, which can result in serious detrimental health effects^19,20^. To minimize the hypercalcemic effect, many efforts are currently being directed to develop biased agonists of VDR that have little effect on inducing hypercalcemia while retaining anticancer activities comparable to those of calcitriol^173–175^. To date, nearly 1500 vitamin D analogs have been tested for such effects, but only a few among those compounds have been approved for further evaluation in clinical trials in patients with leukemia, breast, prostate, and colon cancers^17^. Moreover, the metabolites produced from the CYP11A1-driven alternative vitamin D metabolism pathway have been shown to be a biased agonist of VDR with less calcemic effect while retaining anti-proliferative properties in cancer comparable to those of calcitriol^32,39^. As the alternative vitamin D metabolism pathway via CYP11A1 is just beginning to be understood, its role in cancer and the relative contributions of VDR and ROR are largely unknown. Thus, intensive research on the alternative vitamin D metabolism pathway and successful application of this pathway for cancer therapy is warranted in the future. In addition, increasing local concentrations of calcitriol in cancer cells would be another strategy to avoid the hypercalcemic effect of calcitriol. As a calcitriol degrading enzyme, CYP24A1 is frequently overexpressed in many cancers; the inhibition of CYP24A1 can increase local concentrations of calcitriol in cancer cells^176^. Indeed, recent studies showed that inhibition of CYP24A1 by genetic knockdown or pharmacological inhibition greatly sensitized the anticancer effect of calcitriol^176^. So far, several CYP24A1-specific inhibitors have been developed for clinical purposes and it remains to be seen whether any of these will be used for cancer therapy with suitable clinical effectiveness and safety^176^.

Additionally, because the expression levels of CYP27B1, VDR, CYP11A1, and RORα/γ in cancer cells progressively decline during cancer progression as discussed earlier, the effectiveness of vitamin D-based therapy may be limited to only early stages, but not late stages, of cancer. Thus, identifying and developing novel diagnostic markers predicting the effectiveness of vitamin D-based cancer therapy may be indispensable for the successful application of such a strategy. Moreover, improved understanding of the mechanisms by which cancer progression reduces the expression of those enzymes will be a critical step for successful vitamin D-based cancer therapy. Currently, as discussed above, the mechanisms that regulate the expression and/or activity of metabolic enzymes involved in CYP27B1-VDR and CYP11A1-RORα/γ pathways are poorly understood. Recent advances in understanding cancer metabolism suggest that many oncogenic signaling pathways converge on metabolic pathways by regulating the expression and/or activity of metabolic enzymes^177^. Accordingly, these oncogenic signaling pathways are highly likely to regulate the expression and/or activity of enzymes involved in vitamin D metabolism and function. Unraveling such intricate networks involving the oncogenic and vitamin D metabolism pathways will contribute to the understanding of dysregulated vitamin D metabolism and function in cancer and also provide promising new opportunities for cancer therapy.
